# Unlocking the Determinants of Digital and Technological Self-Efficacy: Insights from a Cross-Sectional Study Among Nurses and Nursing Students

**DOI:** 10.3390/healthcare13172208

**Published:** 2025-09-03

**Authors:** Gianluca Conte, Cristina Arrigoni, Arianna Magon, Giada De Angeli, Giulia Paglione, Irene Baroni, Silvia Belloni, Greta Ghizzardi, Ippolito Notarnicola, Alessandro Stievano, Rosario Caruso

**Affiliations:** 1 Clinical Research Service, IRCCS Policlinico San Donato, 20097 San Donato Milanese, Italy; 2 Department of Public Health, Experimental and Forensic Medicine, University of Pavia, 27100 Pavia, Italy; 3 School of Nursing, Azienda Socio Sanitaria Territoriale di Lodi, 26900 Lodi, Italy; 4 Department of Medicine and Surgery, Kore University of Enna, 94100 Enna, Italy; 5 Department of Clinical and Experimental Medicine, University of Messina, 98122 Messina, Italy; 6 Department of Biomedical Sciences for Health, University of Milan, 20133 Milano, Italy

**Keywords:** digital self-efficacy, digital competency, technology adoption, nursing education, healthcare technology, digital literacy, determinants, predictors, nurses, nursing students

## Abstract

Background: Digital self-efficacy is a crucial determinant of healthcare professionals’ ability to adapt to technological innovations. Understanding its predictors among nurses and nursing students is essential for workforce readiness. Objectives: To assess the level of digital self-efficacy and examine demographic, educational, and experiential factors associated with inadequate self-efficacy. Methods: This cross-sectional study involved 1081 Italian nurses and nursing students. The Digitech-S scale was used to measure digital self-efficacy, with ≥70/100 indicating adequacy. Logistic regression was performed to identify predictors of inadequate self-efficacy. Results: Only 47.1% of participants demonstrated adequate self-efficacy. Females had twice the odds of inadequate self-efficacy compared to males (OR = 2.038, *p* < 0.001). Nurses with bachelor’s degrees had 2.5 times higher odds than students (OR = 2.450, *p* < 0.001), while post-graduate education showed no effect. Early technology adoption before age 14 reduced the odds (OR = 0.675, *p* = 0.027). Each additional year of work experience decreased the odds by 4% (OR = 0.955, *p* < 0.001). Conclusions: Gender disparities persist in digital self-efficacy, and unexpectedly, students outperformed bachelor-level nurses. Findings highlight educational gaps and the importance of early exposure to technology. Tailored interventions are needed to strengthen digital readiness, which may improve care quality and healthcare system efficiency in the digital era.

## 1. Introduction

Digital and Technological Solutions (DTS) are fundamentally reshaping how people experience their everyday lives [1,2,3]. Indeed, the “digital revolution” frequently mentioned in the scientific literature has become a present-day reality for many professionals [4,5,6]. Within this evolving scenario, DTS serve as the foundation for many sectors, with healthcare representing one of the most important [7,8]. Notably, multiple stakeholders in healthcare are and will be responsible for this transformation, with nurses, as the largest professional group, positioned at the forefront of this trend [9,10]. Given this need, nurses are expected to be prepared and fully supported to competently utilize DTS in all its forms [11,12]. It is pivotal that nurses, including nursing undergraduate students, have the knowledge, skills, and resources they need to use DTS at the bedside and in the community, especially since many initiatives related to technological adoption in healthcare fail due to a lack of user uptake [13].

Accordingly, many scholars have developed tools to measure nurses’ and nursing students’ knowledge, skills, and attitudes in this domain [14,15,16,17,18,19,20,21,22,23,24,25]. Recently, a theoretical framework named Digitech-F hypothesized the possible connections among these domains [26]. The Digitech-F offers an integrative view that allows distinguishing different types of competence and capabilities, going beyond traditional computer competence to embrace the wider spectrum of digital capabilities including social media and other digital tools [26]. The framework recognizes that nurses’ competency in DTS extends beyond mere technical skills and considers a range of essential components, including skills, knowledge, attitude, competence, behaviors, individual characteristics, and digital self-efficacy levels [26]. Subsequently, tools that measure digital self-efficacy, like the Digitech-S tool, have been developed and validated [27], with applications in specialized areas such as cardiology being suggested [28].

In this context, digital and technological self-efficacy measured by these tools is defined as an individual’s confidence in proficiently performing digital and technological tasks. Self-efficacy as a concept constitutes the core construct of Bandura’s social cognitive theory, characterized as a dynamic cognitive process that pertains to an individual’s confidence in effectively executing a specific task under challenging circumstances [29,30,31]. Moreover, self-efficacy tends to mediate the relationship between individual-level characteristics (such as knowledge and attitudes regarding DTS) and work performance: low levels of self-efficacy might increase the likelihood of avoiding challenging situations (such as using DTS), which could negatively impact patient outcomes and working performance [32]. However, the literature suggests that despite all the advantages, nurses and many other health professionals occasionally resist adopting healthcare technology [19], indicating that individual characteristics may play crucial roles in determining digital readiness and behaviors.

Specifically, individual characteristics of the professionals represent the first and foundational pillar that determines other kinds of self-efficacy domains [33,34,35]. In fact, before acquiring specific nursing competencies and confidence levels, each person brings unique traits, such as sex, age, and prior experience, that may influence their capabilities. Although the Digitech-F framework has recently conceptualized the multifaceted nature of digital competence and validated tools exist to measure digital self-efficacy, limited research has systematically examined how these individual factors contribute to digital self-efficacy among nurses. Most existing studies have instead focused on general attitudes toward technology or basic computer skills, rather than capturing a comprehensive view of digital self-efficacy using validated instruments. This gap in knowledge undermines the ability of scholars, clinicians, educators, and policymakers to design effective reskilling interventions, which are increasingly urgent in today’s rapidly evolving healthcare environment. Accordingly, this study hypothesizes that individual characteristics significantly influence digital and technological self-efficacy among nurses and nursing students. To address this gap, the present study, referred to as the Digitech-S *delta* substudy, aims to identify and describe the key determinants shaping digital and technological self-efficacy within these populations.

## 2. Materials and Methods

### 2.1. Design

Following established guidelines for self-reported scale design and development [36], a methodological, multi-phase study was conducted and published at the end of 2023 to develop and validate the Digitech-S scale [27]. In early 2025, a secondary analysis was conducted on the same dataset, which included a large sample of Italian nurses and nursing students, to describe the determinants influencing Digital and Technological Self-Efficacy among nurses and nursing students. This study presents the results of this secondary analysis. Of the 1081 participants enrolled in the original study, 955 provided complete Digitech-S data, and 906 had complete data for all study variables and were included in the final multivariate analysis.

### 2.2. Procedure

In the parent study [27], data were collected using a convenience sampling strategy. Registered nurses and undergraduate nursing students from multiple academic and clinical institutions were invited to participate. Recruitment targeted accessible populations within two Italian universities and two teaching hospitals for the first sample and subsequently from a different university and hospital for the second sample. All eligible individuals were invited without exclusion criteria to maximize representativeness across different educational and professional backgrounds.

Also, in the original Digitech-S study, participants were informed about the study’s objectives, data protection measures, and confidentiality before providing electronic informed consent. The primary survey was conducted anonymously with no collection of personally identifiable information. Data were gathered through an online, cloud-based self-report questionnaire, which included demographic variables (e.g., age, sex, education, work setting, clinical experience), self-perceived digital competence, and responses to the Digital and Technological Self-Efficacy Scale (Digitech-S). The electronic survey ensured consistent administration and allowed for secure data collection across multiple sites.

### 2.3. Individual Characteristics

To comprehensively describe the determinants influencing Digital and Technological Self-Efficacy among nurses and nursing students, all relevant individual characteristics from the original sample were retrieved. Specifically, the following variables were collected: age (years), gender (male, female, other), principal occupation (registered nurse, student), education (bachelor’s degree or equivalent qualification, post-graduate education including a master’s degree and PhD, in training and not working for both bachelor’s and advanced degrees), total years of work experience (years), work environment (medical care, surgical care, critical care, other services, or no work history for students), age at which participants first began to regularly use technology (years, also dichotomized as ≤14 years versus >15 years), and self-perceived autonomy in the context of work or education when using digital solutions (requiring some form of support versus completely autonomous).

### 2.4. Digitech-S Scale Characteristics

The primary outcome measure was digital self-efficacy, assessed using the Digitech-S scale [27]. The Digitech-S is a 10-item self-report instrument specifically developed to measure individuals’ confidence in their ability to successfully perform digital and technological tasks in healthcare settings. The scale functions as a “thermometer” of digital self-efficacy, with items arranged hierarchically from basic to more advanced competencies. Respondents rate their confidence for each item using a five-point Likert scale ranging from 1 (“completely unconfident”) to 5 (“completely confident”). The total score is obtained by summing individual item scores, with higher scores indicating higher digital self-efficacy.

The psychometric properties of the Digitech-S were established through a multi-step validation process. Initial item selection employed Mokken Scaling Analysis (MSA), resulting in strong scalability coefficients (Hs = 0.67; Hi ranging from 0.55 to 0.72; rho reliability = 0.95), which demonstrated robust unidimensionality and hierarchical ordering. The scale was further validated using a Multiple-Indicators Multiple-Causes (MIMIC) model, which confirmed the unidimensional structure (χ^2^/df = 1.9, CFI = 0.974, TLI = 0.967, RMSEA = 0.049). This analysis resulted in the removal of non-invariant items to achieve measurement invariance across groups of registered nurses and nursing students. The final version demonstrated high internal consistency (Cronbach’s α = 0.928) and supported configural and metric invariance across groups in a subsequent multi-group confirmatory factor analysis. The complete list of scale items is presented in Table 1.

### 2.5. Ethical Considerations

Ethical approval for the original study protocol, which forms the basis of this secondary analysis, was granted by the local Institutional Review Board (IRB) at the time of the parent study (IRB of the University of Pavia, Reference No. 2/CD/2021, dated 1 February 2021), and permissions for conducting the study and secondary analyses were obtained from all relevant institutions. Furthermore, participants provided electronic informed consent, and data management adhered to the European Union’s General Data Protection Regulation (GDPR 2016/679).

### 2.6. Statistical Analysis

Initially, the collected data were examined to ensure quality and accuracy by systematically reviewing all responses for missing information, unusual values, or data entry errors. Subsequently, categorical variables were described using frequencies and percentages to illustrate the proportion of participants in each category.

Continuous variables (such as age or years of experience) were then examined to determine whether they followed a normal distribution pattern. This assessment was conducted using two mathematical approaches: analysis of skewness and kurtosis, followed by the Shapiro–Wilk test. Accordingly, normally distributed continuous variables were summarized using mean ± standard deviation (SD), which provides the average value and the typical amount of variation around that average.

To identify which individual characteristics were associated with adequate digital self-efficacy, logistic regression (LR) analysis was employed to predict adequate versus inadequate digital self-efficacy. This method allows researchers to determine which factors independently influence the likelihood of achieving adequate self-efficacy while controlling for the effects of other variables simultaneously. LR was preferred over linear regression because it allows for a more granular classification of individuals into adequate and inadequate self-efficacy groups rather than merely explaining the variance of continuous self-efficacy scores. This approach facilitates a more meaningful interpretation of the factors that independently influence the probability of achieving adequate self-efficacy while simultaneously controlling for potential confounders.

Specifically, variables that showed a meaningful relationship with digital self-efficacy were included as predictors in the LR model. These variables were defined as having a point-biserial correlation coefficient with *p*-value ≤ 0.1, indicating at least a weak statistical association [37]. Moreover, the overall performance and reliability of the statistical model were evaluated using two established measures: the Hosmer–Lemeshow test (which assesses how well the model fits the actual data) and Nagelkerke’s pseudo-*R*^2^. The latter indicates what proportion of the variation in digital self-efficacy the model can explain.

Additionally, all predictor variables were entered into the model simultaneously rather than sequentially, allowing the researchers to examine each variable’s unique contribution to predicting digital self-efficacy while accounting for the influence of all other variables. Finally, all statistical analyses were performed using IBM SPSS^®^ Statistics for macOS, version 29 (IBM Corp., Armonk, NY, USA), with statistical significance defined as *p* < 0.05.

## 3. Results

### 3.1. SocioDemographic and Occupational Characteristics of the Sample

A total of 1081 participants were enrolled in this study. The mean age was 32.9 years (SD = 12.7). The sample was predominantly female (*n* = 862; 81.4%), with males representing a smaller proportion. The majority were registered nurses (*n* = 656; 60.7%), while 39.3% (*n* = 425) were nursing students. Regarding educational attainment, 30.0% (*n* = 324) held a bachelor’s degree or equivalent qualification, 28.8% (*n* = 311) had completed post-graduate education (master’s degree, PhD), and 37.9% (*n* = 410) were in training for a bachelor’s degree and not currently working. Among working participants, the mean total years of work experience was 10.1 years (SD = 12.5). The largest proportion of workers were employed in other services (*n* = 250; 23.1%), followed by medical care (*n* = 146; 13.5%) and surgical care (*n* = 137; 12.7%), with 39.3% having no work history (self-identified as students). The mean age at which participants first began to regularly use technology was 16.5 years (SD = 8.1). Regarding self-perceived autonomy in digital solutions within work or educational contexts, 57.4% (*n* = 621) indicated requiring some form of support, while 39.0% (*n* = 422) reported being completely autonomous. Overall, sociodemographic and occupational characteristics are summarized in Table 2.

### 3.2. Defining Predictors in the Logistic Regression Model

Based on Bandura’s recommendations, the Digitech-S scores were standardized to a 0–100 scale [27,29,30,31].To establish a clinically meaningful cutoff for digital self-efficacy, ROC curve analysis was performed using the standardized Digitech-S scores. The optimal cutoff point was determined using Youden’s Index, which maximizes the sum of sensitivity and specificity [38]. Based on this analysis, a standardized score of 70 and above was considered to represent adequate digital self-efficacy, as this threshold provided the optimal balance between sensitivity (66.5%) and specificity (74.6%), with a Youden’s Index of 0.411. This cutoff point ensures reasonable discrimination between individuals with adequate versus inadequate digital self-efficacy while maintaining acceptable classification accuracy for both research and practical applications. Considering the 8.7% missing data in the specific item (the web survey’s most challenging and lengthy section, making dropout expected), among 955 responders, 46.4% (*n* = 443) demonstrated adequate digital self-efficacy.

Bivariate analysis was conducted to identify independent variables for assessing the determinants of adequate digital self-efficacy. Adequate digital self-efficacy (categorized as 1 = adequate and 0 = inadequate) was more frequent among participants who were registered nurses versus students (rpb = 0.131; *p*-value < 0.001), had higher education levels, with postgraduate education (master’s degree, PhD) coded as higher than bachelor’s degree or equivalent qualifications (rpb = 0.194; *p*-value < 0.001), and reported higher scores of autonomy in technology use (rpb = 0.410; *p*-value < 0.001). Conversely, inadequate digital self-efficacy was more frequent in participants of older age (rpb = −0.241; *p*-value < 0.001), among females compared to males (rpb = −0.159; *p*-value < 0.001), greater total years of work experience (rpb = −0.246; *p*-value < 0.001), working in clinical contexts versus no work history (rpb = −0.088; *p*-value = 0.006), and higher age at first technology use (rpb = −0.263; *p*-value < 0.001). All variables demonstrated *p*-values ≤ 0.1 and were therefore included in subsequent multivariate analysis. Complete bivariate analysis results, including correlation coefficients and significance levels, are presented in Table 3.

### 3.3. Determinants Influencing Digital and Technological Self-Efficacy

Of the 1081 participants enrolled in this study, 906 had 100% complete questionnaire data, including all necessary variables for inclusion in the model (a complete case response rate of 83.8%). The Hosmer–Lemeshow test showed good model fit (*p* = 0.450), indicating effective relationships between variables and the outcome. The model explained 16.1% of the variance in digital self-efficacy (Nagelkerke *R*^2^ = 0.161).

The likelihood of achieving adequate digital self-efficacy was significantly enhanced by starting daily technology use before age 14, which reduced the odds of inadequate self-efficacy by 32% (Adj. OR = 0.675; 95% CI = 0.477–0.955; *p* = 0.027), and by each additional year of work experience, which reduced the odds of inadequate self-efficacy by 4% (Adj. OR = 0.955; 95% CI = 0.938–0.973; *p* < 0.001).

Conversely, inadequate digital self-efficacy was more likely among females, who showed twice the odds of inadequate self-efficacy compared to males (Adj. OR = 2.038; 95% CI = 1.409–2.948; *p* < 0.001), and among nurses with only a bachelor’s degree, who had 2.5 times higher odds of inadequate self-efficacy compared to students (Adj. OR = 2.450; 95% CI = 1.664–3.607; *p* < 0.001). No significant differences were observed between nurses with postgraduate education and nursing students (*p* = 0.204). Principal occupation (registered nurse or student) and work environment showed no significant associations. Complete multivariate LR results are presented in Table 4.

## 4. Discussion

This study provided a comprehensive analysis of the determinants of Digital and Technological Self-Efficacy among Italian nurses and nursing students. Among these determinants, several individual characteristics showed significant effects in predicting adequate digital self-efficacy levels. The findings reveal that targeting interventions to address key predictors, particularly for female healthcare professionals and both inexperienced and older nurses, could bridge existing gaps, fostering a generation of digitally confident and skilled nursing professionals.

The most striking finding was the substantial gender disparity in digital self-efficacy, with females showing twice the odds of inadequate self-efficacy compared to males. This finding is particularly concerning given that nursing is a predominantly female profession (81.4% in the sample), suggesting that the majority of the nursing workforce may be at risk for lower digital self-efficacy. These gender disparities may be understood through the lens of digital capital theory, which extends Bourdieu’s concept of cultural capital to the digital realm [39]. Digital capital encompasses not only access to digital technologies but also the skills, knowledge, and social support necessary for effective technology use [40]. Research suggests that women may have differential access to digital capital due to sociocultural factors, including gendered expectations about technology competence [41] and varying levels of encouragement in STEM-related fields [42]. In nursing, a predominantly female profession, these broader societal patterns may be amplified, creating systemic barriers to digital self-efficacy development. These findings align with established technology acceptance frameworks. Also, according to UTAUT theory, individual characteristics such as gender and age influence technology acceptance through performance expectancy and social influence mechanisms [43]. The gender disparities observed may reflect differential performance expectancy beliefs, where sociocultural factors influence women’s confidence in technology outcomes. This gender gap represents a critical challenge that requires immediate attention, as it could perpetuate existing inequalities in healthcare technology adoption and utilization.

Educational background emerged as another significant determinant in this study, with nurses holding only a bachelor’s degree demonstrating 2.5 times higher odds of inadequate digital self-efficacy compared to nursing students. This paradoxical finding may reflect differences in social and cultural capital related to digital technologies. Current nursing students, predominantly from Generation Z, have grown up with ubiquitous digital technologies and possess what Helsper and Eynon (2010) term ‘digital nativity’: not merely early exposure, but immersion in digital culture [44]. This generational cohort benefits from peer networks that normalize technology use and educational environments that increasingly integrate digital tools [45]. Conversely, nurses educated in earlier digital eras may lack these supportive social networks and normalized technology experiences, resulting in lower digital self-efficacy despite greater clinical experience. The finding that nursing students showed comparable digital self-efficacy to nurses with postgraduate education further supports this interpretation, as postgraduate education often involves intensive technology use and digital scholarship, potentially compensating for earlier educational gaps. From a technology acceptance perspective, these patterns align with Davis’ Technology Acceptance Model, which suggests that perceived ease of use is influenced by individual experience and training [46]. Early digital exposure may enhance perceived ease of use, while later adoption may be hindered by initial difficulty perceptions.

Furthermore, the temporal aspects of technology exposure in life proved crucial, with early technology adoption (before age 14) significantly reducing the odds of inadequate digital self-efficacy. This finding underscores the importance of early digital socialization and suggests that the ‘digital native’ concept [47] has practical implications for nursing workforce development, consistent with research showing generational differences in technology comfort and adoption patterns [48,49]. Early technology adoption represents a form of cultural capital acquisition during formative years when individuals develop fundamental attitudes and competencies toward technology. Those who begin regular technology use before age 14 benefit from extended periods of skill development and confidence building during critical developmental stages [50]. In this regard, Van Dijk’s digital access model provides additional insight: while material access (technology availability) may be uniform in healthcare settings, motivational access (interest and confidence) and skills access (digital competencies) clearly vary by demographic characteristics, ultimately affecting usage access (effective application in practice) [51].

Finally, the relationship between work experience and digital self-efficacy presents a complex picture: while each additional year of experience reduced inadequate self-efficacy by 4%, this relationship likely reflects the confounding effects of age and generational differences in technology adoption. From a social capital perspective, experienced nurses may benefit from workplace learning networks and mentorship relationships that facilitate gradual technology adoption over time [52]. However, this positive effect must be weighed against potential age-related barriers to technology acceptance that have been documented in healthcare settings [53]. Also, according to the Diffusion of Innovations theory, workplace exposure and peer networks can accelerate technology adoption, which may explain why experienced nurses in supportive environments demonstrate improved digital self-efficacy over time [54].

### 4.1. Implications for Practice and Policy

The identification of both modifiable and non-modifiable determinants has important implications for multiple stakeholders. For nurse educators, these findings suggest the need for targeted digital literacy curricula that address gender-specific barriers and provide intensive support for female nursing students. This recommendation aligns with existing literature documenting persistent gender gaps in STEM education [42] and technology confidence in nursing programs [55,56]. Research has identified that nursing curricula often inadequately address digital competencies [12] and may inadvertently perpetuate gender-based technology anxieties through traditional pedagogical approaches [57]. Educational programs should focus on building digital capital by incorporating early and continuous exposure to digital technologies, ensuring that all graduates achieve adequate digital self-efficacy regardless of their background in technology.

For healthcare organizations and policymakers, the results highlight the urgent need for tailored professional development programs. Female nurses, those with basic educational backgrounds, and those who began using technology later in life should be prioritized for digital skills training. Additionally, mentorship programs pairing digitally confident nurses with those requiring support could leverage social capital mechanisms while addressing skill gaps. Such programs should recognize that digital self-efficacy development requires not only technical training but also the cultivation of supportive social networks and confidence-building experiences, consistent with previous frameworks emphasis on social influence and facilitating conditions [43].

Then, the research community should focus on understanding the mechanisms underlying the observed disparities through theoretical lenses of capital accumulation and technology acceptance models. Future investigations should explore how organizational culture, peer networks, and educational interventions can systematically build digital capital among nursing professionals. Moreover, empirical studies are needed to test the effectiveness of targeted training programs designed to address the specific determinants identified in this study.

### 4.2. Modifiable Determinants and Strategic Interventions

Among the determinants identified, several are modifiable through strategic interventions. Educational background, while partially fixed, can be enhanced through continuing education and professional development programs. Digital autonomy in workplace settings represents a highly modifiable factor that organizations can address through supportive policies, mentorship programs, and gradual skill-building initiatives.

The workplace context, although not significant in the final model, remains an important consideration for intervention design. Organizations should create environments that support digital experimentation and learning, reducing the fear of technology adoption that may disproportionately affect certain demographic groups in an era where artificial intelligence (AI), other than classical computer and internet skills, emerges as the new revolution in the nursing discipline [51].

### 4.3. Study Limitations and Future Directions

This substudy has some limitations that should be acknowledged. The cross-sectional design limited to Italy prevents causal inferences and generalization to other healthcare systems or cultural contexts where different patterns of digital capital distribution may exist. The convenience sampling approach of the original study, while practical, may limit the representativeness of the findings. Future research should employ longitudinal designs to understand how digital self-efficacy and associated capital forms evolve over time and how interventions can modify these trajectories.

The model presented in the study explained only 16.1% of variance in digital self-efficacy, indicating that unmeasured factors likely play important roles. The model primarily included demographic and relatively stable individual characteristics, but did not assess actual digital competencies, technology anxiety, or contextual workplace factors that may significantly influence self-efficacy. Future research should incorporate direct measures of digital skills and competencies alongside self-efficacy assessments to better understand the relationship between objective abilities and confidence levels.

Also, the Digitech-S scale does not explicitly capture competencies related to AI, which is increasingly integral to contemporary healthcare environments. As AI tools become embedded in clinical decision-making, electronic health records, and patient care systems, future research should develop measures that encompass AI-specific digital self-efficacy alongside traditional digital competencies. Future investigations should incorporate these theoretical constructs to provide a more comprehensive understanding of the mechanisms driving digital self-efficacy development in nursing.

Furthermore, cross-national research will be essential to determine whether the observed patterns of capital distribution and digital self-efficacy are universal or context-specific. The role of cultural factors, educational systems, and healthcare technology infrastructure may significantly influence the relationships between individual characteristics and digital capital accumulation across different countries and healthcare settings. Such research should explicitly incorporate technology acceptance frameworks and digital access models to provide comprehensive theoretical grounding for international comparisons.

## 5. Conclusions

The findings of the Digitech-S *delta* substudy reveal disparities in digital self-efficacy among Italian nurses and nursing students, with gender, educational background, and early technology exposure serving as key determinants. These results provide crucial insights into the mechanisms driving digital readiness disparities within the nursing workforce. The identification of gender as the strongest predictor, with females showing twice the odds of inadequate digital self-efficacy, represents a critical challenge given nursing’s predominantly female composition, while the paradoxical finding that current nursing students outperform bachelor’s degree nurses highlights generational differences that suggest traditional nursing education may inadequately prepare graduates for contemporary digital healthcare demands.

Addressing these determinants through theoretically informed interventions could substantially improve the digital behaviors of the nursing workforce, ultimately enhancing patient care quality and healthcare system efficiency in an increasingly digital healthcare environment. The identification of modifiable factors provides a roadmap for evidence-based interventions that can bridge existing digital divides and prepare nursing professionals for the technological future of healthcare delivery.

## Figures and Tables

**Table 1:** Digitech-S items.

	ITALIAN VERSION	ENGLISH VERSION
	*Pensando alla Sua esperienza lavorativa e all’utilizzo della tecnologia nel Suo contesto, Le chiediamo di indicare quanto si sente capace di affrontare le seguenti situazioni nel quotidiano.*	*Thinking about your work experience and the use of technology in your context, we ask you to indicate how capable you feel in handling the following situations in your daily life.*
1	Acquisire in un tempo ristretto nuove conoscenze in ambito tecnologico, considerando che molte tecnologie cambiano velocemente	Acquire new knowledge in technology in a short time, considering that many technologies change rapidly
2	Trovare risposte a situazioni sfidanti che derivano dalla mia pratica lavorativa sfruttando i motori di ricerca e gli applicativi che utilizzano Internet (web-based)	Finding answers to challenging situations that arise from my work practice using search engines and applications that use the internet (web-based)
3	Acquisire nuove conoscenze seguendo corsi o seminari totalmente online	Gain new knowledge by following full-online courses or seminars
4	Acquisire dimestichezza con nuovi applicativi o tecnologie, prediligendo un apprendimento incentrato sull’utilizzo pratico piuttosto che sul “prendere appunti”	Become familiar with new applications or technologies, preferring learning strategies focused on practical use rather than on “taking notes”
5	Pianificare una curva di auto-apprendimento per migliorare le mie competenze digitali e tecnologiche	Plan a self-learning curve to improve my digital and technological skills
6	Inviare una e-mail con allegati molti file	Send an e-mail containing numerous file attachments
7	Avviare una videochiamata entro pochi minuti con gli strumenti di cui dispongo nel mio contesto	Start a video call within minutes with the tools I have at my disposal
8	Restare aggiornato con le nuove versioni di applicativi, servizi digitali e dispositivi	Stay up-to-date with new versions of applications, digital services, and devices
9	Collaborare efficacemente in team anche utilizzando mezzi digitali (esempio, videochiamata, e-mail ecc.)	Effectively collaborate in teams while also utilizing digital tools (example, video calls, e-mail, etc.)
10	Vincere un eventuale pregiudizio nei confronti del mondo della tecnologia (esempio, efficacia della formazione a distanza)	Overcoming any preconception about the tech world (example, the effectiveness of distance learning)

**Table 2:** Characteristics of the sample (*n* = 1081 †).

		*n*	%
Age			
	Years (mean, SD)	32.9	12.7
Gender			
	Male	197	18.6
	Female	862	81.4
	Other	1	N/A
Principal occupation			
	Registered nurses	656	60.7
	Student	425	39.3
Education			
	Bachelor’s degree or equivalent qualification	324	30.0
	Post-graduate education (master’s degree, PhD)	311	28.8
	In training and not working (bachelor’s degree)	410	37.9
	In training and not working (master’s degree, PhD)	15	1.4
Total years of work *			
	Years (mean, SD)	10.1	12.5
Work environment			
	Medical care	146	13.5
	Surgical care	137	12.7
	Critical care	95	8.8
	Other services	250	23.1
	No work history (self-identified as a student)	453	41.9
Age at which they first begin to regularly use technology			
	Years (mean, SD)	16.5	8.1
Self-perceived autonomy, in the context of work or education, when using digital solutions			
	Some form of support is required	621	57.4
	Completely autonomous	422	39.0

Notes: † Missing data account for up to 6.5% in some variables, with sample sizes ranging from 1081 to 1010. * Only workers were assessed for this variable. Legend: SD: standard deviation.

**Table 3:** Point-biserial correlation coefficients between adequate digital self-efficacy and predictor variables.

**Variable**	**rpb**	***p*** **-Value**	**Inclusion**
Age	−0.241	<0.001	Included
Gender	−0.159	<0.001	Included
Principal occupation (RN or student)	0.131	<0.001	Included
Education	0.194	<0.001	Included
Total years of work	−0.246	<0.001	Included
Work environment (if applicable)	−0.088	0.006	Included
Age at first technology use	−0.263	<0.001	Included
Self-perceived autonomy	0.410	<0.001	Included

Notes: All variables with *p* ≤ 0.1 were included in subsequent analyses. N ranges from 902 to 955, depending on missing data for each variable. Legend: rpb = point-biserial correlation coefficient; RN = registered nurse.

**Table 4:** Determinants of adequate Digital and Technological Self-Efficacy (*n* = 906).

	Adj. OR	95% CI	*p*-Value
**Gender (female vs. male)**	2.038	1.409–2.948	<0.001 *
**Principal occupation (RN or student)**	1.156	0.742–1.801	0.518
**Education**			
**Bachelor’s degree nurses**	2.450	1.664–3.607	<0.001 *
**Postgraduate nurses**	1.341	0.852–2.111	0.204
**Total years of work**	0.955	0.938–0.973	<0.001 *
**Work environment (if applicable)**	0.923	0.651–1.307	0.651
**Age at first daily technology use** **(≥14 years vs.** **<14 years)**	0.675	0.477–0.955	0.027 *

* Statistically significant.

## Data Availability

The data presented in this study are not publicly available due to ethical and privacy restrictions related to participant confidentiality. However, de-identified data may be made available from the corresponding author upon reasonable request and with appropriate institutional and ethical approvals.
